# Supplementary material for: M2 macrophages independently promote beige adipogenesis via blocking adipocyte Ets1
Source: Nat Commun. 2024 Feb 22;15:1646. doi: 10.1038/s41467-024-45899-4 (PMC10883921; doi:10.1038/s41467-024-45899-4)
Supplement: Supplementary file 1 — Supplementary Information [file 41467_2024_45899_MOESM1_ESM.pdf]

Supplementary information for

M2 macrophages independently promote beige adipogenesis via blocking adipocyte Ets1

Authors： Suyang Wu[1, 5], Chen Qiu[1, 3, 5], Jiahao Ni[1], Wenli Guo[1], Jiyuan Song[1], Xingyin Yang[1], Yulin Sun[1], Yanjun Chen[1], Yunxia Zhu[1], Xiaoai Chang[1], Peng Sun[1], Chunxia Wang[4], Kai Li[1, 2*], Xiao Han[1, *]

Affiliations:

1 Key Laboratory of Human Functional Genomics of Jiangsu Province, Department of Biochemistry and Molecular Biology, Nanjing Medical University, Nanjing 211166, China

2 Department of Endocrinology, The Affiliated Taizhou People's Hospital of Nanjing Medical University, Taizhou School of Clinical Medicine, Nanjing Medical University, Taizhou 225300, China

3 Key Laboratory of the Model Animal Research, Animal Core Facility of Nanjing Medical University, Nanjing 211166, China

4 Laboratory of Critical Care Translational Medicine, Institute of Pediatric Infection, Immunity, and Critical Care Medicine, Shanghai Jiao Tong University School of Medicine, Shanghai, 200062, China

5 These authors contributed equally to this work.

* Correspondence: likai87@njmu.edu.cn; hanxiao@njmu.edu.cn. Phone Num. +86 15251709941

This PDF file includes：

Fig. S1 to S6
Table S1 to S3

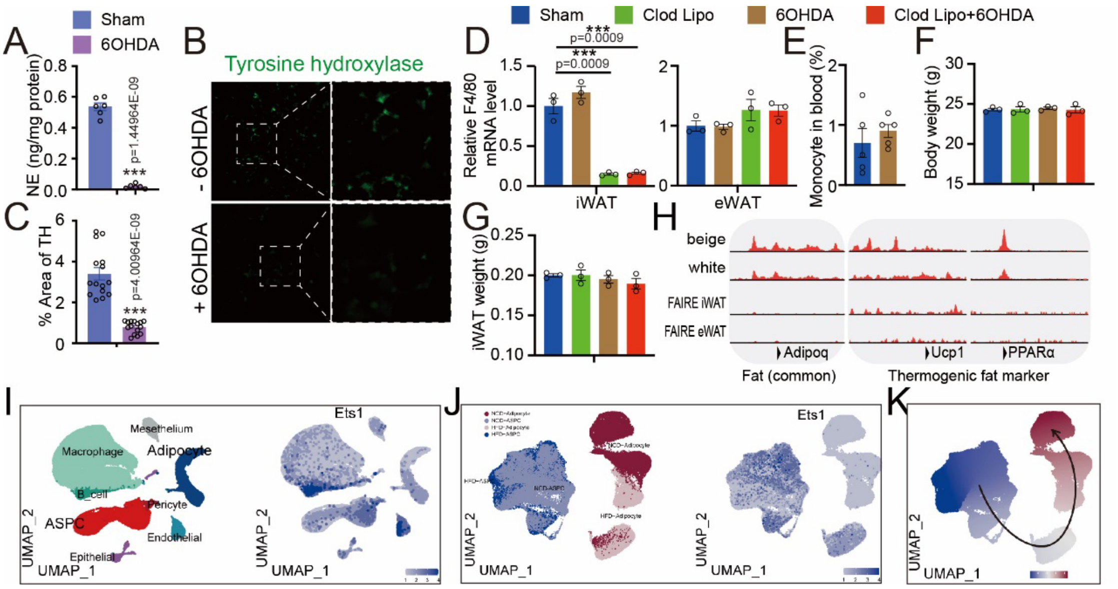

Supplementary Figure 1: Macrophages directly regulate the adipocyte beiging process independent of the sympathetic nervous system.

A-C: Wild type C57/BL6j mice received intraperitoneal injection of 6-OHDA (100 mg/kg) (n=6) or vehical (n=6), on day -5, -3, and -1. At day 4, the blocking effect of 6OHDA on the sympathetic nervous of iWAT were analyzed.

A: Norepinephrine content normalized to total protein content in iWAT.

B: Immunofluorescence staining of sympathetic marker tyrosine hydroxylase (Th) in iWAT.

C: Quantification of percentage TH density in iWAT sections (2-3 fields of view per mouse).

D-G: All the four group of sham, 6OHDA alone, Clod Lipo alone, and 6OHDA+Clod Lipo were cold stressed for 4 days, started from Day 0.

D: Quantitative PCR testing the expression of macrophage marker F4/80 in iWAT. N=3 per group.

E: Monocyte number in circulating blood, measured by blood cell analysis. N=6 per group.

F: Body weight of the mice after cold stress. N=3 per group.

G: Inguinal adipose tissue weight of the mice after cold stress. N=3 per group.

H: The IGV tool visualization of the open peaks on the promoter region of adipocyte marker genes. SVFs separated from iWAT of wild type C57 mice were induced differentiation towards beige or white adipocyte for 7 days, then analyzed by ATAC-seq. A published FAIRE-seq dataset of brown and white adipose (GSE83764) were together analyzed and treated as control.

I-K: Analyzing the correlation of Ets1 with adipocyte differentiation, using a published single cell transcription sequencing dataset (GSE176171).

I: UMAP and unsupervised clustering of high fat diet fed mice adipose tissue (left). The expression of Ets1 across these cells were profiled (right).

J: UMAP projection of only the ASPC and mature adipocytes of both chow diet and high fat diet fed mice (left). The expression of Ets1 across these cells were profiled (right).

K: Pseudotime differential trajectory analysis of ASPC and mature adipocytes, inferred by the Slingshot method. Cells are color-coded for defined pseudotime score.

Data are means ± SEM. Two-sided Student's t test was used to evaluate statistical significance.

Source data are provided as a Source Data file.

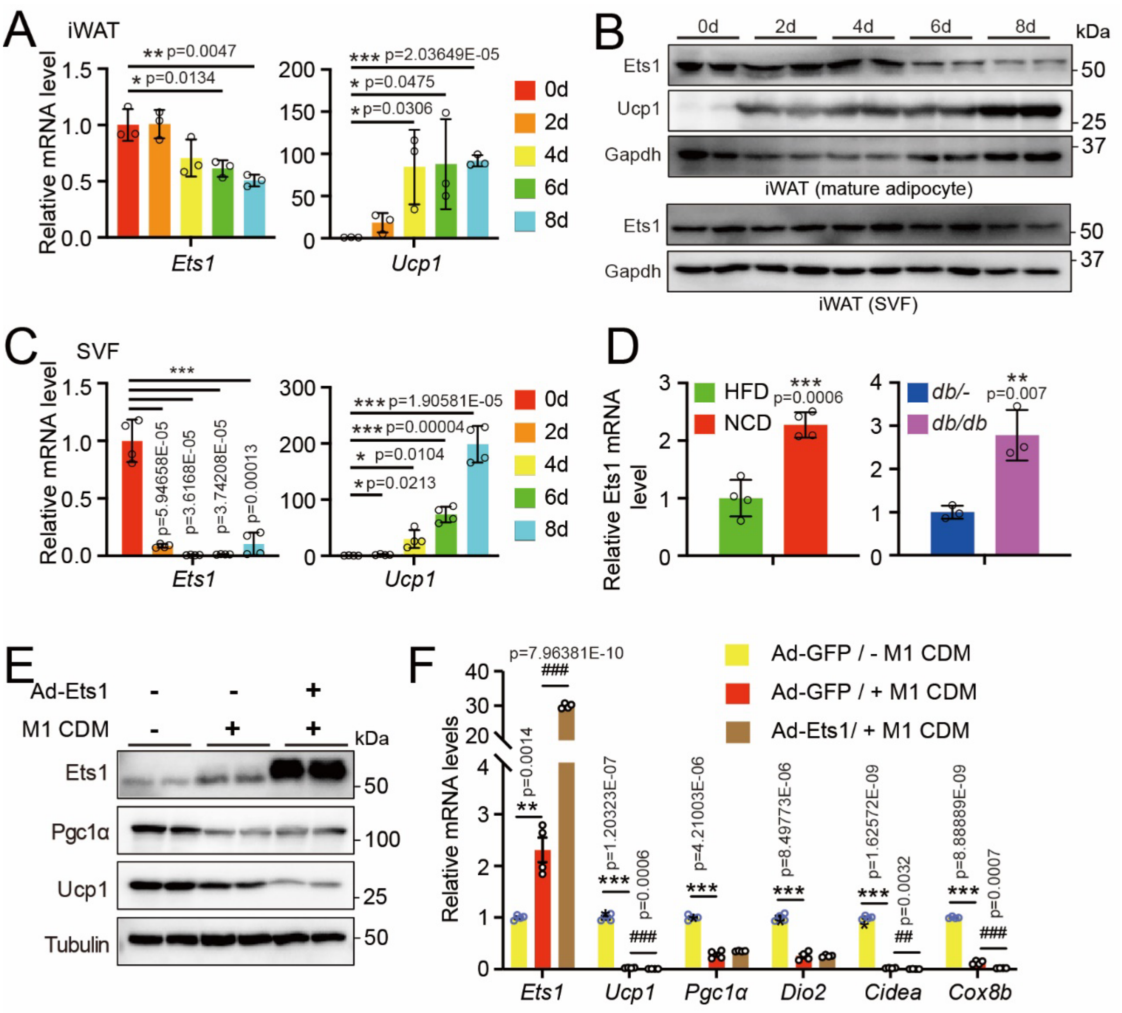

Supplementary Figure 2: Ets1 negatively associated with beige adipogenesis.

A: Quantitative PCR showing the mRNA levels of Ets1 and thermogenic marker Ucp1 in iWAT at 0, 2, 4, 6, and 8 days after cold stress. n=3 per group.

B: Western blot showing the protein levels of Ets1 in mature adipocyte (up) or in SVF cells (down) derived from iWAT, at 0, 2, 4, 6, and 8 days after cold stress. n=2 per group.

C: SVFs separated from wild type C57 were differentiated into beige adipocytes. At indicated time point after differentiation, mRNA of Ets1 and Ucp1 were analyzed by qPCR. n=4 per group.

D: Quantitative PCR showing the mRNA levels of Ets1 in mature adipocyte derived from iWAT. Left: High-fat diet (HFD) and normal chow diet (NCD) feedings were started at 4 weeks of age and continued for 16 weeks. Right: *db/-* and *db/db* mice were 8-10 weeks old. n=4 per group.

E-F: SVFs separated from iWAT of wild type C57 mice were cultured *in vitro*. The plate-fully-covered cells were infected with indicate adenovirus for 6h, then induced differentiation towards beige adipocytes. 5 days later, the cells were treated with 50% M1 CDM for 24h.

E: Western blot showing the protein levels of Ets1 and thermogenic markers. n=2 per group.

F: Quantitative PCR showing the mRNA levels of Ets1 and thermogenic markers. n=4 per group.

All blot assay was repeated two times independently. Data are means ± SEM. Two-sided Student's t test was used to evaluate statistical significance. Source data are provided as a Source Data file.

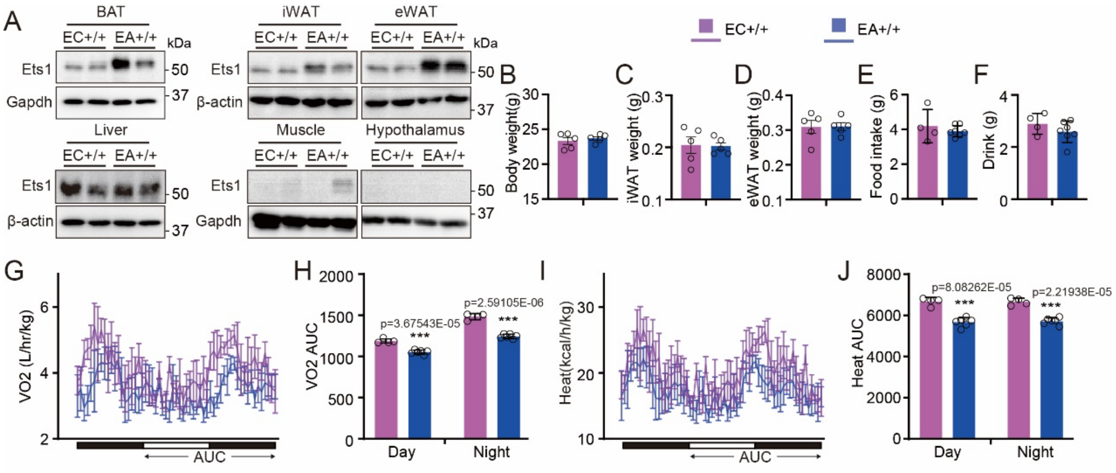

Supplementary Figure 3: Adipocyte-specific Ets1 knock-in mice are cold intolerance.

A: Immunoblotting assay showing the protein levels of Ets1 in all three fat pad types and other metabolism-related organs. Ets1$^{ki/ki}$ mice, abbreviated as EC+/+; adipoq-cre × Ets1 $^{ki/ki}$ mice, abbreviated as EA+/+. N=2 per group.

B-J: EC+/+ and EA+/+ were intraperitoneally treated with Cl316243 for 7 days. On day 5, the mice were transferred into the home-cage system, and metabolic parameters from day 7 to day 9 were analyzed. The black and white bars represent night and day, respectively, and the arrow areas were calculated.

B: Body weight at day 9. n=5 per group.

C: Inguinal adipose tissue weight at day 9. n=5 per group.

D: Epididymal adipose tissue weight at day 9. n=5 per group.

E: Daily food intake. n=4 for EC+/+, n=7 for EA+/+.

F: Daily drink intake. n=4 for EC+/+, n=7 for EA+/+.

G: Oxygen consumption level. n=4 for EC+/+, n=6 for EA+/+.

H: Calculated AUC of D.

I: Heat generation. n=4 for EC+/+, n=6 for EA+/+.

J: Calculated AUC of F.

All blot assay was repeated two times independently. Data are means ± SEM. Two-sided Student's t test was used to evaluate statistical significance. Source data are provided as a Source Data file.

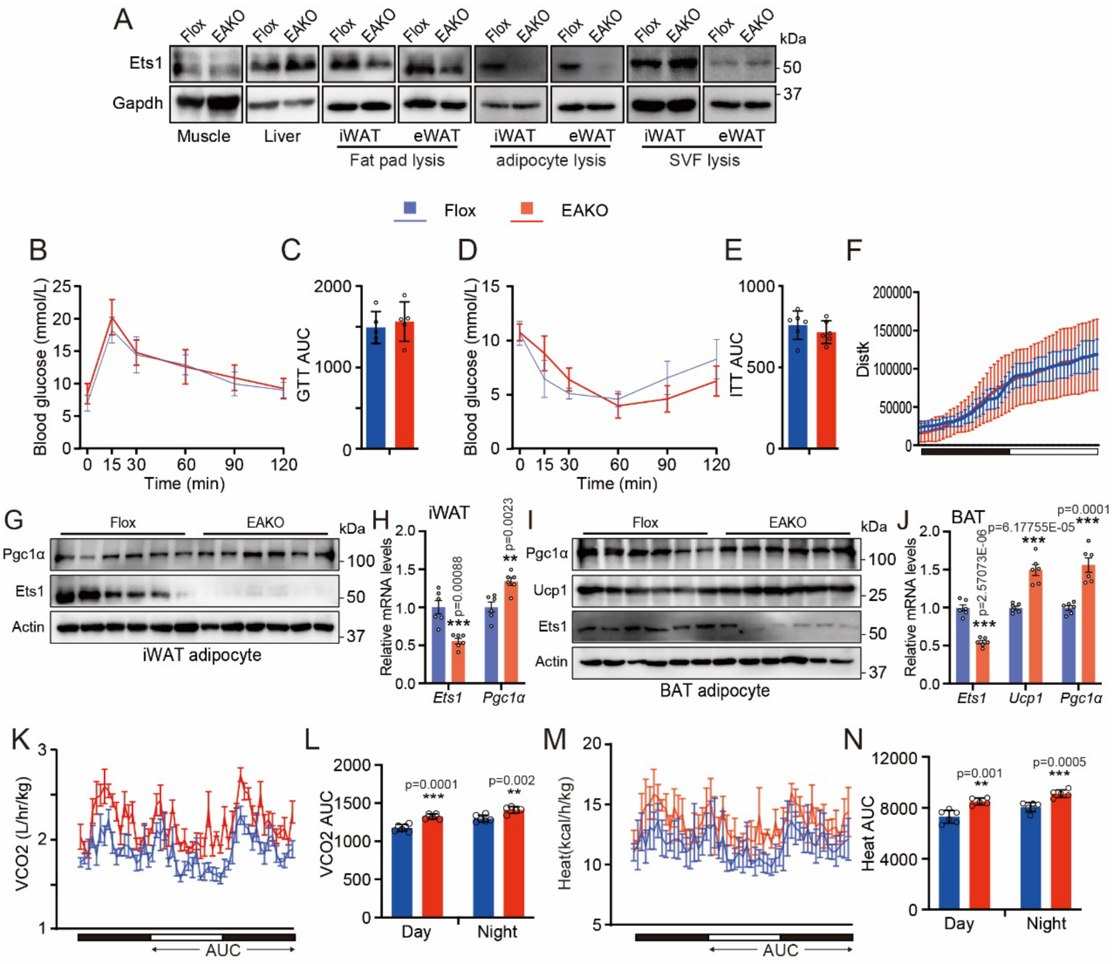

Supplementary Figure 4: Adipocyte Ets1 knock-out mice are resistant to HFD-induced obesity.

A-F: Ets1 f/f (Flox) and adipocyte-specific knock-out (EAKO) mice fed a normal chow diet (NCD) were used.

A: Western blot analysis of Ets1 protein levels in whole fat pads, in mature adipocyte, in SVF cell, and in other metabolic organs.

B: GTT assay. N=5 per group.

C: Calculated AUC of B.

D: ITT assay. N=6 per group.

E: Calculated AUC of E.

F: Locomotor activity. N=6 per group.

G-N: After 14 weeks of HFD feeding, the metabolic parameters of Flox and EAKO mice were analyzed using a home-cage system. The black and white bars represent night and day, respectively, and the arrow areas were calculated.

G-J: Analyzing the expression of thermogenic marker gene either in protein level (G, I), or in mRNA level, of iWAT (G-H) and eWAT (I-J). n=6 per group

K: Oxygen consumption level. n=6 for Flox, n=5 for EAKO.

L: Calculated AUC of D. n=6 for Flox, n=5 for EAKO.

M: Heat generation. n=6 for Flox, n=5 for EAKO.

N: Calculated AUC of F. n=6 for Flox, n=5 for EAKO.

All blot assay was repeated two times independently. Data are means ± SEM. Two-sided Student's t test was used to evaluate statistical significance. Source data are provided as a Source Data file.

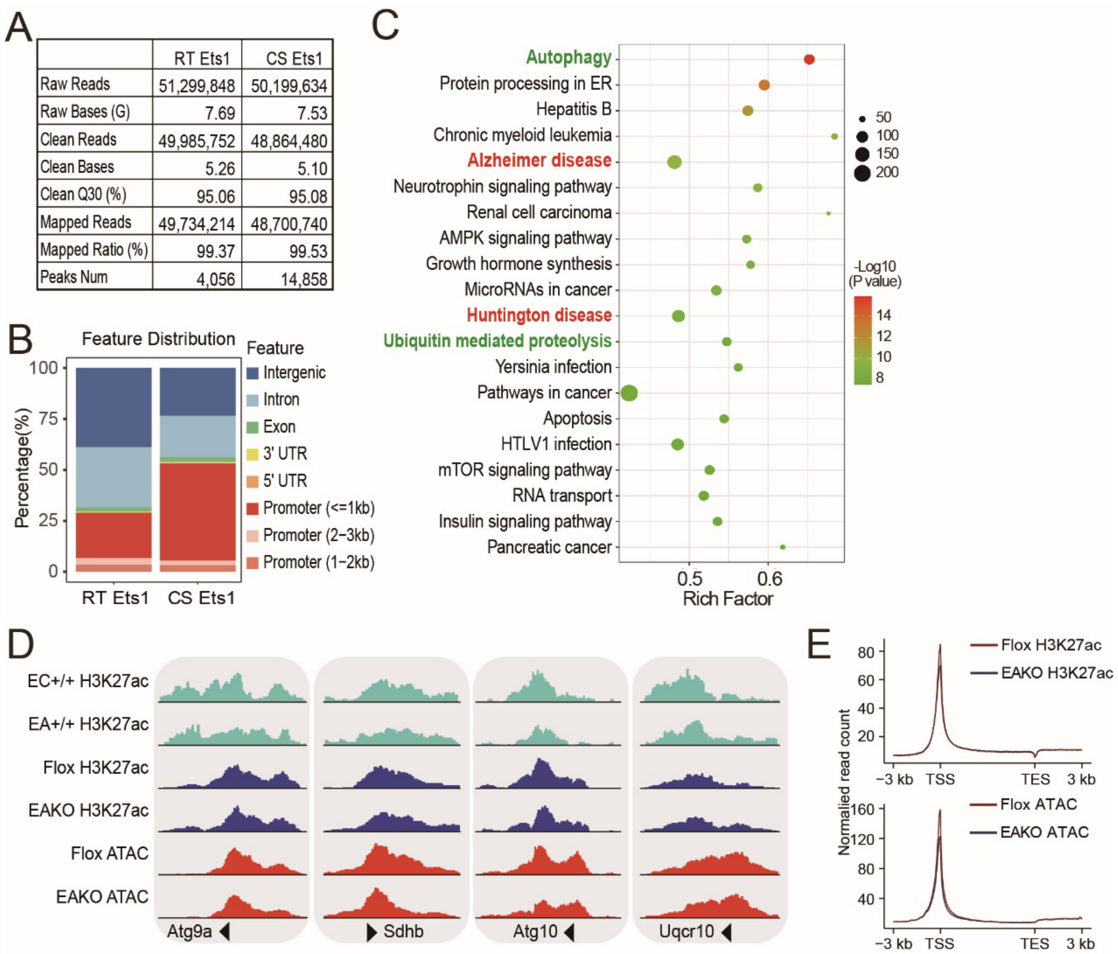

Supplementary Figure 5: ChIP-seq and ATAC-seq data analysis.

A-C: Wild type 8- to 10-week-old C57/BL6j mice were kept either at room temperature (RT) or under cold stress (CS) for 7 days. Mature adipocytes separated from iWAT were used for ChIP-seq (Cut & Tag). N=1 per group.

A: The depth and quality of sequencing.

B: Percentages of peak distributions.

C: KEGG enrichment analysis of genes closest to the peaks of the 1132 down regulated peaks in figure 4C. Mitochondria complex coding genes subcategories in Alzheimer disease, Huntington disease, and autophagy-related genes subcategories in Autophagy, Ubiquitin mediated proteolysis were highlighted.

D-E: Mature adipocytes separated from iWAT of EA+/+, EAKO, and their corresponding control littermates were analyzed by epigenetic sequencing. Histone 3 lys 27 acetylation (H3K27ac) ChIP-seq and assays for transposase-accessible chromatin (ATAC) sequencing were performed. N=1 per group.

D: The IGV tool was used to visualize the binding peaks of Ets1 on the promoter region of autophagy-related and mitochondrial genes.

E: Normalized enrichment of H3K27ac modifications (up) and ATAC-Seq peaks (down) across the gene body (average of reads signal across all genes). TSS, transcription start site; TES, transcription end site.

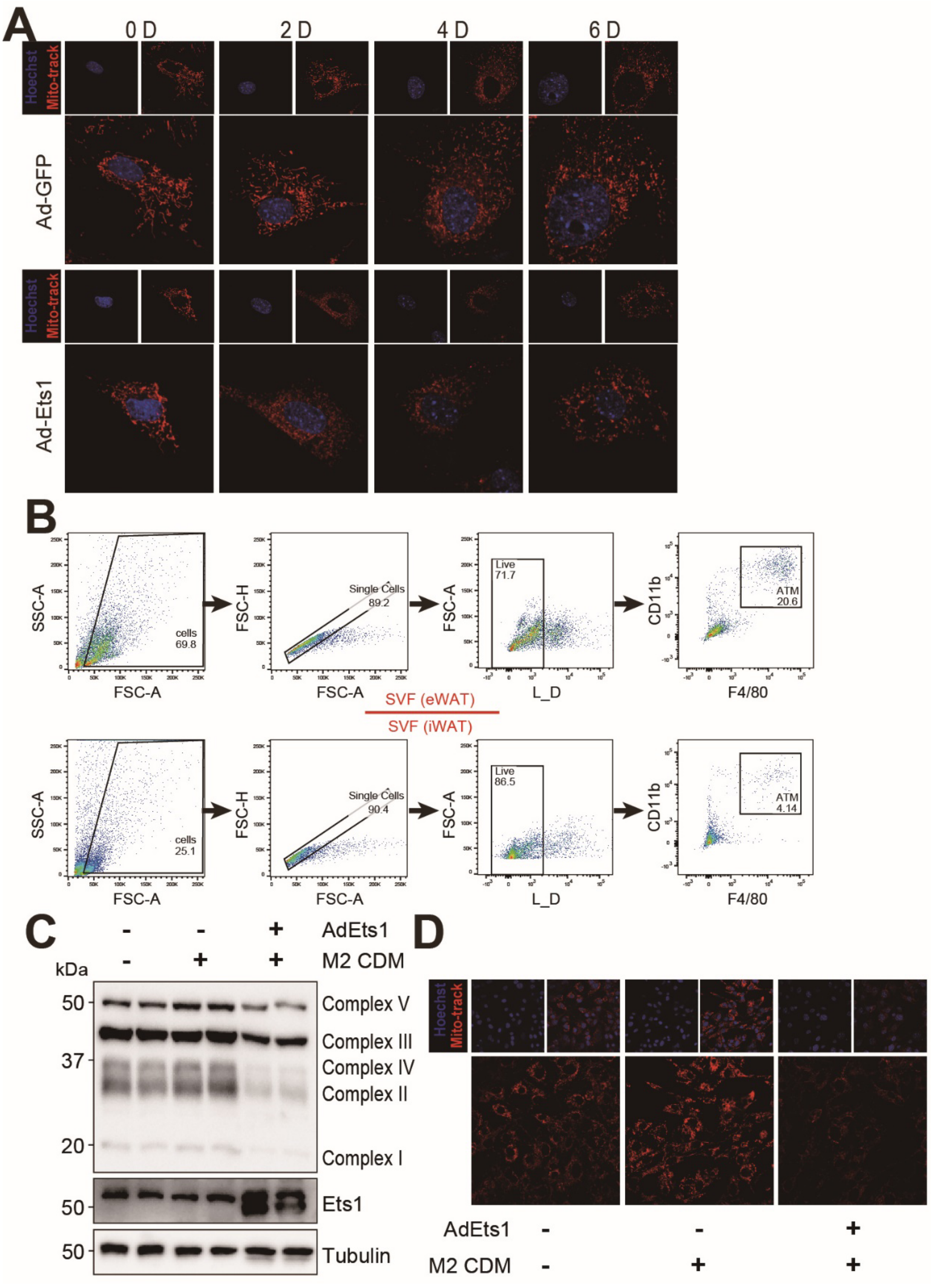

Supplementary Figure 6: M2 macrophage boosts adipocyte mitochondria content via blocking Ets1.

A: Representative images of Mito-track immunofluorescence at days 0, 2, 4, and 6 after beige adipocyte differentiation (n=10 per group). Nuclei were stained with Hoechst (in blue).

B: Adipose tissue macrophage (ATM) separation. Wild type C57/BL6j mice were cold stressed for 48h, the F4/80$^+$, CD11b$^+$ ATMs from both iWAT and eWAT were sorted via flow cytometry, collected and cultured in dishes to harvest ATM CDM.

C-D: SVFs separated from iWAT of wild type C57 mice were cultured *in vitro*. The plate-fully-

covered cells were infected with indicate adenovirus for 6h, then induced differentiation towards beige adipocytes. 5 days later, the cells were treated with 100% M2 CDM for 24h. n=2 for each group.

C: Western blot analysis of the level of OXPHOS and Ets1. The assay was repeated two times independently.

D: Mito-track staining of differentiated adipocyte, showing the mitochondria content.

Supplementary Tables

Supplementary Table 1: Primer sequences for genotyping PCR (5'-3')

Gene	Forward	Reverse
Ets1-KI-WT	CTCTACTGGAGGAGGACAAACTG	GTCTTCCACCTTTCTTCAGTTAGC
Ets1-KI-Mut	GCATCTGACTTCTGGCTAATAAAG	GTCTTCCACCTTTCTTCAGTTAGC
Ets1-loxp	TTGCAGTCACATGTATGGGATAGG	GTAGGCACCCTACAGTACCACAGAG
Adipoq-cre	CGTATAGCCGAAATTGCCAG	CAAAACAGGTAGTTATTCGG

Supplementary Table 2: Antibodies information

**Name**	**Source**	**Manufacturer**	**Catalog**	**Dilution**
Ets1	Rabbit	Cell Signaling Technology, Danvers, MA, USA	#14069	1:1000
p-ERK1/2 (Thr202/Tyr204)	Rabbit	Cell Signaling Technology, Danvers, MA, USA	#4370	1:1000
ERK1/2	Rabbit	Cell Signaling Technology, Danvers, MA, USA	#4695	1:1000
LC3A/B	Rabbit	Cell Signaling Technology, Danvers, MA, USA	#12741	1:1000
Beclin-1	Rabbit	Cell Signaling Technology, Danvers, MA, USA	#3495	1:1000
Pink1	Rabbit	Cell Signaling Technology, Danvers, MA, USA	#6946	1:1000
Parkin	Mouse	Cell Signaling Technology, Danvers, MA, USA	#4211	1:1000
Pgc1α	Rabbit	Abcam, Cambridge, UK	ab188102	1:1000
Ucp1	Rabbit	Abcam, Cambridge, UK	ab209483	1:1000
Mito complex cocktail	Mouse	Abcam, Cambridge, UK	ab110413	1:1000
Hdac1	Rabbit	ABclonal, Wuhan, China	A19571	1:1000
P62	Rabbit	Proteintech, Wuhan, China	18420-1-AP	1:1000
Tubulin	Mouse	Cell Signaling Technology, Danvers, MA, USA	#2148	1:4000
β-actin	Mouse	Cell Signaling Technology, Danvers, MA, USA	#3700	1:4000
Gapdh		Bioworld, Nanjing, China	H200115	1:8000

Supplementary Table 3: Primer sequences for qRT-PCR

Gene	Forward	Reverse
Ets1	TCCTATCAGCTCGGAAGAACTC	TCTTGCTTGATGGCAAAGTAGTC
Ucp1	CACCTTCCCGCTGGACACT	CCCTAGGACACCTTTATACCTAATGG
Dio2	CAGTGTGGTGCACGTCTCCAATC	TGAACCAAAGTTGACCACCAG
Cidea	ATCACAACTGGCCTGGTTACG	TACTACCCGGTGTCCATTTCT
Cox8b	GAACCATGAAGCCAACGACT	GCGAAGTTCACAGTGGTTCC
Pgc1α	AGCCGTGACCACTGACAACGAG	GCTGCATGGTTCTGAGTGCTAAG
Drp1	TTACGGTTCCCTAAACTTCACG	GTCACGGGCAACCTTTTACGA
Mfn1	CCTACTGCTCCTTCTAACCCA	AGGGACGCCAATCCTGTGA
Opa1	TGGAAAATGGTTCGAGAGTCAG	CATTCCGTCTCTAGGTTAAAGCG
Pink1	TTCTTCCGCCAGTCGGTAG	CTGCTTCTCCTCGATCAGCC
Parkin	TTTCTGCCGGGACTGTAAGG	TGCCATCCAATTAGCTTTCC
Ndufv2	GCAAGGAATTTGCATAAGACAGC	TAGCCATCCATTCTGCCTTTG
Sdhb	AATTTGCCATTTACCGATGGGA	AGCATCCAACACCATAGGTCC
Uqcr10	ATCCCTTCGCGCCTGTACT	GTGCTCGTAGATCGCGTCT
Cox8a	TGTGGGGATCTCAGCCATAGT	AGTGGGCTAAGACCCATCCTG
Atp5a1	TCTCCATGCCTCTAACACTCG	CCAGGTCAACAGACGTGTCAG
TFAM	ATTCCGAAGTGTTTTTCCAGCA	TCTGAAAGTTTTGCATCTGGGT
Nrf1	AGCACGGAGTGACCCAAAC	TGTACGTGGCTACATGGACCT
Nrf2	TCTTGGAGTAAGTCGAGAAGTGT	GTTGAAACTGAGCGAAAAAGGC
Atg2a	CCACCTCTGCAAATCGGCA	CCAGTTGTCCTGATACCTCCA
Atg4b	TATGATACTCTCCGGTTTGCTGA	GTTCCCCCAATAGCTGGAAAG
Atg4d	GTCAAGTATGGTTGGGCAGTT	TGTCACCCTCTCCCTCGAAAT
Atg9a	CAGTTTGACACTGAATACCAGCG	AATGTGGTGCCAAGGTGATTT
Ulk1	AAGTTCGAGTTCTCTCGCAAG	CGATGTTTTCGTGCTTTAGTTCC
Atg16L2	GCAGCTTGTGCAGCGTAAG	CTGGTTGGCCCTCTCTCTAC
