# Supplementary material for: M2 macrophages independently promote beige adipogenesis via blocking adipocyte Ets1
Source: Nat Commun. 2024 Feb 22;15:1646. doi: 10.1038/s41467-024-45899-4 (PMC10883921; doi:10.1038/s41467-024-45899-4)
Supplement: Supplementary file 2 — Reporting Summary [file 41467_2024_45899_MOESM2_ESM.pdf]

nature portfolio

Corresponding author(s): Kai Li ; Xiao Han

Last updated by author(s): Dec 28, 2023

Reporting Summary

Nature Portfolio wishes to improve the reproducibility of the work that we publish. This form provides structure for consistency and transparency in reporting. For further information on Nature Portfolio policies, see our Editorial Policies and the Editorial Policy Checklist.

Statistics

For all statistical analyses, confirm that the following items are present in the figure legend, table legend, main text, or Methods section.

n/a	Confirmed	
☐	☒	The exact sample size (*n*) for each experimental group/condition, given as a discrete number and unit of measurement
☐	☒	A statement on whether measurements were taken from distinct samples or whether the same sample was measured repeatedly
☐	☒	The statistical test(s) used AND whether they are one- or two-sided<br>*Only common tests should be described solely by name; describe more complex techniques in the Methods section.*
☒	☐	A description of all covariates tested
☐	☒	A description of any assumptions or corrections, such as tests of normality and adjustment for multiple comparisons
☐	☒	A full description of the statistical parameters including central tendency (e.g. means) or other basic estimates (e.g. regression coefficient) AND variation (e.g. standard deviation) or associated estimates of uncertainty (e.g. confidence intervals)
☐	☒	For null hypothesis testing, the test statistic (e.g. *F*, *t*, *r*) with confidence intervals, effect sizes, degrees of freedom and *P* value noted<br>*Give P values as exact values whenever suitable.*
☒	☐	For Bayesian analysis, information on the choice of priors and Markov chain Monte Carlo settings
☒	☐	For hierarchical and complex designs, identification of the appropriate level for tests and full reporting of outcomes
☒	☐	Estimates of effect sizes (e.g. Cohen's *d*, Pearson's *r*), indicating how they were calculated

Our web collection on statistics for biologists contains articles on many of the points above.

Software and code

Policy information about availability of computer code

Data collection	Western- ChemiScope 3100(Clinx)(Version 7.1.2.0)<br>qPCR- LightCycler 48011 (Roche)<br>Imaging- FV1200 confocal microscope(Olympus); DM IRB fluorescent microscope(Leica)<br>BWA<br>Macs2<br>deeptools<br>ChIPseeker<br>DEGseq
Data analysis	Fiji (Image J bundle, version 2.9.0); GraphPad Prism (version 10); FV10 ASW 4.2 Viewer; Seahorse Wave Pro(Version 10.1.0.1); TSE PhenoMaster(mouse)(Version 4.5.3)

For manuscripts utilizing custom algorithms or software that are central to the research but not yet described in published literature, software must be made available to editors and reviewers. We strongly encourage code deposition in a community repository (e.g. GitHub). See the Nature Portfolio guidelines for submitting code & software for further information.

Data

Policy information about availability of data

All manuscripts must include a data availability statement. This statement should provide the following information, where applicable:
- Accession codes, unique identifiers, or web links for publicly available datasets
- A description of any restrictions on data availability
- For clinical datasets or third party data, please ensure that the statement adheres to our policy

All data from the study are available in the main text or the supplementary materials. The generated raw datasets, together with the analyzed bigwig and narrowpeak files during the current study are available in the GEO repository GSE221335 and GSE234879.

Research involving human participants, their data, or biological material

Policy information about studies with human participants or human data. See also policy information about sex, gender (identity/presentation), and sexual orientation and race, ethnicity and racism.

Reporting on sex and gender	N/A
Reporting on race, ethnicity, or other socially relevant groupings	N/A
Population characteristics	N/A
Recruitment	N/A
Ethics oversight	N/A

Note that full information on the approval of the study protocol must also be provided in the manuscript.

Field-specific reporting

Please select the one below that is the best fit for your research. If you are not sure, read the appropriate sections before making your selection.

☒ Life sciences ☐ Behavioural & social sciences ☐ Ecological, evolutionary & environmental sciences

For a reference copy of the document with all sections, see nature.com/documents/nr-reporting-summary-flat.pdf

Life sciences study design

All studies must disclose on these points even when the disclosure is negative.

Sample size	The sample size for the animal experiments was not calculated. For animal experiments, we tried our best to ensure that the experimental mice were of the same week age. Therefore, according to the number of newborn mice, the number of each experimental group was about 4-7, which was detailed in the figure legend. For confocal images quantification, 300 cells from 3 independent trials were counted. For Flow cytometry analysis, 6000 cells from 3 independent trials were analyzed. For qPCR analysis or other analysis, 3 independent trials were performed. The sample size was chosen based on the suggestions from the reviewers and previous studies in the field. We included as many animals as possible in the experiments in order to achieve high statistical significance, taking into consideration factors such as variance and survival rates. We also took into account similar studies when determining the sample sizes.
Data exclusions	No data exclusions
Replication	All experiments, except for sequencing approaches were conducted with a minimum of two independent experiments. All replication attempts indicated similar results. As for sequencing, the ATAC-seq was conducted with two biological replications in each group, while the Ets1/H3k27ac ChIP-seq were performed with no replication.
Randomization	Not performed since all groups received the same conditions, within the remit of control or experimental conditions. There was no differential treatment between groups on the basis of genotypes or other biological distinctions, and thus randomisation was deemed to be unnecessary.
Blinding	We used Single-blinding. Two different investigators were involved as follows: One investigator grouped the animals, treated the cells and conducted the experimental operation, and the other collected experimental data and statistical analysis.

Reporting for specific materials, systems and methods

We require information from authors about some types of materials, experimental systems and methods used in many studies. Here, indicate whether each material, system or method listed is relevant to your study. If you are not sure if a list item applies to your research, read the appropriate section before selecting a response.

Materials & experimental systems

n/a	Involved in the study	
☐	☒	Antibodies
☐	☒	Eukaryotic cell lines
☒	☐	Palaeontology and archaeology
☐	☒	Animals and other organisms
☒	☐	Clinical data
☒	☐	Dual use research of concern
☒	☐	Plants

Methods

n/a	Involved in the study	
☐	☒	ChIP-seq
☐	☒	Flow cytometry
☒	☐	MRI-based neuroimaging

Antibodies

Antibodies used

Ets1 Rabbit Cell Signaling Technology, #14069 1:1000
LC3A/B Rabbit Cell Signaling Technology, #12741 1:1000
Beclin-1 Rabbit Cell Signaling Technology, #3495 1:1000
Pink1 Rabbit Cell Signaling Technology, #6946 1:1000
Parkin Mouse Cell Signaling Technology, #4211 1:1000
Tubulin Mouse Cell Signaling Technology, #2148 1:4000
β-actin Mouse Cell Signaling Technology, #3700 1:4000
Pgc1α Rabbit Abcam, ab188102 1:1000
Ucp1 Rabbit Abcam, ab209483 1:1000
OXPHOS Cocktail Mouse Abcam, ab110413 1:1000
Hdac1 Rabbit ABclonal, A19571 1:1000
P62 Rabbit Proteintech, 18420-1-AP 1:1000
Gapdh Bioworlde, MB001H 1:8000
Fcγ receptor block Biolegend 101319 1:100
Fixable Viability Dye Biolegend 423101 1:100
PE-anti-F4/80 Biolegend 123110 1:100
APC-anti-CD11b Biolegend 101212 1:100

Validation

Ets1 Rabbit Cell Signaling Technology, #14069
https://www.cellsignal.cn/products/primary-antibodies/ets-1-d8o8a-rabbit-mab/14069

LC3A/B Rabbit Cell Signaling Technology, #12741

https://www.cellsignal.cn/products/primary-antibodies/lc3a-b-d3u4c-xp-rabbit-mab/12741

Beclin-1 Rabbit Cell Signaling Technology, #3495
https://www.cellsignal.cn/products/primary-antibodies/beclin-1-d40c5-rabbit-mab/3495

Pink1 Rabbit Cell Signaling Technology, #6946
https://www.cellsignal.cn/products/primary-antibodies/pink1-d8g3-rabbit-mab/6946

Parkin Mouse Cell Signaling Technology, #4211
https://www.cellsignal.cn/products/primary-antibodies/parkin-prk8-mouse-mab/4211

Tubulin Mouse Cell Signaling Technology, #2148
https://www.cellsignal.cn/products/primary-antibodies/a-b-tubulin-antibody/2148

β-actin Mouse Cell Signaling Technology, #3700
https://www.cellsignal.cn/products/primary-antibodies/b-actin-8h10d10-mouse-mab/3700

Pgc1α Rabbit Abcam, ab188102
https://www.abcam.cn/products/primary-antibodies/pgc1-alpha--beta-antibody-epr18289-ab188102.html

Ucp1 Rabbit Abcam, ab209483
https://www.abcam.cn/products/primary-antibodies/ucp1-antibody-epr20381-ab209483.html

OXPHOS Cocktail Mouse Abcam, ab110413
https://www.abcam.cn/products/panels/total-oxphos-rodent-wb-antibody-cocktail-ab110413.html

Hdac1 Rabbit ABclonal, A19571
https://abclonal.com.cn/catalog/A19571

P62 Rabbit Proteintech, 18420-1-AP
https://www.ptglab.com/products/SQSTM1-Antibody-18420-1-AP.htm

Gapdh Bioworlde, MB001H
https://www.bioworlde.com/Primary-Antibodies/141071.html

Fcγ receptor block Biolegend 101319
https://www.biolegend.com/en-us/products/trustain-fcx-anti-mouse-cd16-32-antibody-5683

Fixable Viability Dye Biolegend 423101
https://www.biolegend.com/en-us/products/zombie-aqua-fixable-viability-kit-8444

PE-anti-F4/80 Biolegend 123110
https://www.biolegend.com/en-us/products/pe-anti-mouse-f4-80-antibody-4068

APC-anti-CD11b Biolegend 101212
https://www.biolegend.com/en-us/products/apc-anti-mouse-human-cd11b-antibody-345

Eukaryotic cell lines

Policy information about cell lines and Sex and Gender in Research

Cell line source(s)	3T3-L1 (Cat# CL-173), 293T (Cat# CRL-3216) and Cos-7 (Cat #CRL-1651) cell lines were purchased from ATCC<br>ADSCs were derived from iWAT and BAT of 4-week-old C57BL/6 male mice<br>BMDMs were derived from bones of 8-week-old C57BL/6 male mice<br>ATMs were derived from iWAT and eWAT of 12-week-old C57BL/6 male mice
Authentication	No further authentication of the cell lines was performed before use.
Mycoplasma contamination	All cell lines tested negative for mycoplasma contamination.
Commonly misidentified lines (See ICLAC register)	No commonly misidentified lines were used.

Animals and other research organisms

Policy information about studies involving animals; ARRIVE guidelines recommended for reporting animal research, and Sex and Gender in Research

Laboratory animals

All the used mice were C57BL/6j background. Mice were housed in temperature- (20-24°C) and humidity- (30%-70%) controlled, 12:12h light-cycled conventional animal quarters. But in the cold stress assay, only the temperature was changed to 4° C .

Ets1 conditional knock-in mice was constructed by Cyagen Biosciences (Suzhou, China) with C57BL/6J background. Briefly, the gRNA to Hipp11 locus, the donor vector containing “CAG-loxp-Stop-loxp-mouse Ets1 CDS (without stop codon) -2A-EGFP-polyA” cassette, and Cas9 mRNA were co-injected into fertilized mouse eggs to generate targeted conditional knockin offspring. After confirming correctly targeted ES clones, selecting some clones for blastocyst microinjection, followed by chimera production. Founders were confirmed as germline-transmitted via crossbreeding with Flp-deleter.

Ets1 conditional knockout mice was generated at Model Animal Research Center of Nanjing University (Nanjing, China) at the background of C57/BL6J. Exons 2-7 of Ets1 locus were floxed with loxP sites.

The obtained Ets1 knock-in /out mice were propagated with adipoq-cre mice (#028020, Jackson Lab) to obtain the adipocytes specific knock-in and knock-out mice. Homozygous Ets1 adipocytes knock-in mice (Ets1ki/ki * adipoq-cre, EA+/+) were compared with homozygous control littermates (Ets1ki/ki, EC+/+), while heterozygous Ets1 adipocytes knock-in mice (Ets1ki/- * adipoq-cre, EA+/-) were compared with heterozygous control ones (Ets1ki/-, EC+/-). Ets1 adipocytes knock-out mice (Ets1f/f * adipoq-cre, EAKO) were compared with control flox mice (Ets1f/f, Flox).

C57BL/6J mice were purchased from Animal Core Facility of Nanjing Medical University, db/db mice were purchased from the Model Animal Research Center of Nanjing University (MARC, Nanjing, China).

Wild animals	No wild animals were used in the study.
Reporting on sex	Sex as a biological variable was not taken into consideration in this study. Since the metabolism of female mice is affected by the menstrual cycle, we initially focused on male mouse models. As a result, we explicitly stated in the abstract, methods, and results sections that the research was exclusively conducted using male mice.
Field-collected samples	No field-collected samples were used in the study.

Ethics oversight | Nanjing Medical University IACUC

Note that full information on the approval of the study protocol must also be provided in the manuscript.

Plants

Seed stocks | N/A

Novel plant genotypes | N/A

Authentication | N/A

ChIP-seq

Data deposition

- [x] Confirm that both raw and final processed data have been deposited in a public database such as GEO.
- [ ] Confirm that you have deposited or provided access to graph files (e.g. BED files) for the called peaks.

Data access links
May remain private before publication.

https://www.ncbi.nlm.nih.gov/geo/query/acc.cgi?acc=GSE234879
https://www.ncbi.nlm.nih.govfgeo/query/acc.cgi?acc=GSE221335

Files in database submission

GSE221335 RT_1.bw
GSE221335 CS_1.bw
GSE221335 Eco_ H3K27ac.bw
GSE221335 Eki_ H3K27ac.bw
GSE221335 WT_CP.bw
GSE221335 KO_CP.bw
GSE221335 WT.bw
GSE221335 KO.bw
GSE221335 Beg_1.bw
GSE234879 Begl.bw
GSE234879 Beg2.bw
GSE234879 Whtl.bw
GSE234879 Wht2.bw

Genome browser session
(e.g. UCSC)

N.A

Methodology

Replicates | N.A

Sequencing depth

Sample ID Reads Maped Maped% Peaks Number
GSE221335 RT_1.bw 48,931,189 48,700,740 99.53 4056
GSE221335 CS_1.bw 50,050,242 49,734,214 99.37 14858
GSE221335 Eco_ H3K27ac.bw 47,381,989 47,312,789 99.85 33713
GSE221335 Eki_ H3K27ac.bw 56,762,602 56,707,955 99.9 38484
GSE221335 WT_CP.bw 48,447,062 48,410,824 99.93 86834
GSE221335 KO_CP.bw 48,118,146 48,084,468 99.93 74682
GSE221335 WT.bw 92,621,413 91,806,914 99.12 88977
GSE221335 KO.bw 84,901,802 84,110,719 99.07 82839
GSE221335 Beg_1.bw 61,196,700 57,364,064 98.93 30553
GSE234879 Begl.bw 94,780,558 66,663,815 70.33 118812
GSE234879 Beg2.bw 94,549,094 71,989,489 76.14 127490
GSE234879 Whtl.bw 96,575,188 92,642,132 95.93 120473
GSE234879 Wht2.bw 100,729,062 96,579,960 95.88 119385

Antibodies

Ets1, Active Motif, #39581, 1:50;
H3K27ac, Active Motif, #91193, 1:50;
normal rabbit IgG, Merck Millipore #12-370;
normal mouse IgG, Merck Millipore #12-371)
second antibodies:

	anti-rabbit IgG antibody, goat monoclonal: Millipore AP132;<br>anti-mouse IgG antibody, goat polyclonal: Millipore AP124
Peak calling parameters	Prior to mapping pair-end reads, clean reads were acquired from the original reads by eliminating the adapter sequences utilizing the trimmomatic software (Version 0.36). The clean reads were aligned to reference mm10 genome sequences using BWA program (Version 0.7.17). We used macs2 (Version 2.2.8) to call peaks and selected peaks with cutoff q value < 0.05 . The bam file generated by the unique mapped reads as an input file, using deeptools software (Version 3.2.1) for bigwig generation. The deeptools tool (Version 3.2.1) is used for plot the reads distributions (from bigwig) across peaks, followed by annotating the peaks by the function of annotatePeak of ChIPseeker (Version 3.18). The HOMER's tool (Version 4.10) was used for Motif analysis, while the IGV tool was employed for visualization. DEGseq (Version 3.18) was used for identify differentially expressed genes from RNA-seq data.
Data quality	We followed ENCODE guideline for quality control and confirmed high quality (>50 million uniquely reads per sample).
Software	Trimmomatic (Version 0.36, default parameter); BWA (Version 0.7.17, default parameter); Macs2 (Version 2.2.8, default parameter); Deeptools (Version 3.2.1, parameter "-q 1-F 1024"); ChIPseeker (Version 3.18, default parameter). HOMER's tool (Version 4.10, default parameter); DEGseq (Version 3.18, log2 Fold Change>1, Pvalue<0.05); IGV tool.

Flow Cytometry

Plots

Confirm that:

- [x] The axis labels state the marker and fluorochrome used (e.g. CD4-FITC).
- [x] The axis scales are clearly visible. Include numbers along axes only for bottom left plot of group (a 'group' is an analysis of identical markers).
- [x] All plots are contour plots with outliers or pseudocolor plots.
- [x] A numerical value for number of cells or percentage (with statistics) is provided.

Methodology

Sample preparation	SVFs isolated from mice iWAT and eWAT were resuspended in PBS to remove the blood and grease, and Centrifugated at 500 g for 5min. the SVFs were incubated with RBC lysis buffer for 5min and neutralized RBC lysis buffer by adding PBS followed by centrifugation at 500 g for 5 min and resuspension in PBS. The SVFs were incubated with Fcγ receptor block for 20 min at 4 °C before staining. SVF pellets were washed with PBS and stained with Fixable Viability Dye. Cells were washed again and resuspended in PBS. stain with indicated fluorescent-conjugated antibodies for 30min at 4°C in the dark. The antibodies used for the isolation of macrophages included PE-anti-F4/80 and APC-anti-CD11b.
Instrument	Cell sorting was performed with BD FACSAria™ Fusion.
Software	Fluorescence signals were analyzed using FlowJo 10.8.1 software.
Cell population abundance	We stained isolated SVFs by FACS with F4/80, CD11b antibodies. Our flow cytometry analysis revealed that we could obtain ~20% F4/80+CD11b+ macrophages in eWAT and ~4% F4/80+CD11b+ iWAT SVFs.
Gating strategy	Identify singlet and viable cells from SVF, then gate F4/80 positive and CD11b positive cell populations

- [x] Tick this box to confirm that a figure exemplifying the gating strategy is provided in the Supplementary Information.
